# To start or to complete? – Challenges in implementing tuberculosis preventive therapy among people living with HIV: a mixed-methods study from Karnataka, India

**DOI:** 10.1080/16549716.2019.1704540

**Published:** 2020-01-15

**Authors:** Mahendra M. Reddy, Pruthu Thekkur, Nagesh Ramya, Prasanna B. T. Kamath, Suresh G. Shastri, Ravi B. N. Kumar, Palanivel Chinnakali, Abhay S. Nirgude, Chethana Rangaraju, Narasimhaiah Somashekar, Ajay M. V. Kumar

**Affiliations:** a Department of Community Medicine, Sri Devaraj Urs Medical College (SDUMC), Sri Devaraj Urs Academy of Higher Education and Research (SDUAHER), Kolar, India; b Centre for Operational Research, International Union Against Tuberculosis and Lung Disease (The Union), Paris, France; c Centre for Operational Research, The Union South-East Asia Office, New Delhi, India; d Department of Health and Family Welfare Services, State Tuberculosis Cell, Bengaluru, India; e Department of Health and Family Welfare Services, National AIDS Control Organization (NACO), New Delhi, India; f Department of Health and Family Welfare Services, Karnataka AIDS Prevention Society (KSAPS), Bengaluru, India; g Department of Preventive and Social Medicine, Jawaharlal Institute of Postgraduate Medical Education and Research (JIPMER), Puducherry, India; h Department of Community Medicine, Yenepoya Medical College, Yenepoya (Deemed to be University), Mangaluru, India; i National Tuberculosis Institute, Bengaluru, India

**Keywords:** Adherence, latent tuberculous infection, operational research, SORT IT, TB/HIV co-infection

## Abstract

**Background**: Isoniazid preventive therapy (IPT) has been shown to reduce the risk of tuberculosis (TB) among people living with HIV (PLHIV). In 2017, India began a nationwide roll-out of IPT, but there is a lack of evidence on the implementation and the challenges.

**Objectives**: Among PLHIV newly initiated on antiretroviral therapy (ART) from January 2017 to June 2018, to: (i) assess the proportion who started and completed IPT and (ii) explore reasons for non-initiation and non-completion from health-care providers’ and patients’ perspectives.

**Methods**: An explanatory mixed-methods study was conducted in two selected districts of Karnataka, South India. A quantitative phase (cohort analysis of routinely collected program data) was followed by a qualitative phase involving thematic analysis of in-depth interviews with providers (n = 22) and patients (n = 8).

**Results**: Of the 4020 included PLHIV, 3780 (94%) were eligible for IPT, of whom, 1496 (40%, 95% CI: 38%-41%) were initiated on IPT. Among those initiated, 423 (28.3%) were still on IPT at the time of analysis. Among 1073 patients with declared IPT outcomes 870 (81%, 95% CI: 79%-83%) had completed the six-month course of IPT. The main reason for IPT non-initiation and non-completion was frequent drug stock-outs. This required health-care providers to restrict IPT initiation in selected patient subgroups and earmark six-monthly courses for each patient to ensure that, once started, treatment was not interrupted. The other reasons for non-completion were adverse drug effects and loss to follow-up.

**Conclusion**: The combined picture of ‘low IPT initiation and high completion’ seen in our study mirrors findings from other countries. Drug stock-out was the key challenge, which obliged health-care providers to prioritize ‘IPT completion’ over ‘IPT initiation’. There is an urgent need to improve the procurement and supply chain management of isoniazid.

## Background

Tuberculosis (TB) is the most frequent opportunistic infection and the leading cause of mortality among people living with HIV (PLHIV) [1]. In 2017, out of 0.9 million HIV-related deaths globally, about 0.3 million were due to TB [2]. India is home to about 2.1 million PLHIV with a prevalence of 0.2% among adults aged 15–49 years [3]. The reasons for high mortality include failure to suspect and diagnose TB, delays in diagnosis and treatment of TB and missed opportunities for prevention.

To reduce the burden of TB among PLHIV, the World Health Organization (WHO) recommends three interventions: i) intensified TB case finding (ICF); ii) TB prevention using isoniazid preventive therapy (IPT) and early antiretroviral therapy (ART), irrespective of cluster of differentiation 4 (CD4) cell count or clinical staging, and iii) infection control in HIV care facilities and other congregate settings [4,5]. Implementing all these interventions is essential to end the TB epidemic as envisaged in the END TB strategy of the WHO and the Sustainable Development Goals of the United Nations [6,7].

As the adage ‘prevention is better than cure’ goes, preventing TB is much better than trying to detect and treat it, both from an individual patient and a public health perspective. Early ART enrollment is a potent TB prevention tool among PLHIV – both at the individual and community levels [8,9]. There has been impressive progress in global access to ART, with about 21.7 million people receiving ART in 2017 [10]. But is ART alone enough? Unfortunately, the answer is no. While ART greatly reduces the risk of TB, the risk never falls to levels seen in HIV-negative individuals [11,12]. So, we need additional interventions to achieve optimal TB prevention. IPT is one such proven intervention and has been shown to reduce the risk of TB in both adults and children [13–15]. Many randomized controlled trials (RCTs) and observational studies have demonstrated that IPT and ART have a synergistic effect on TB prevention and reduction of mortality [16–20].

Although the WHO has recommended the use of IPT since 2004, its implementation remains poor [21]. Only 36% of all the newly diagnosed PLHIV globally in 2017 were reported to have started on IPT [2]. The other challenge relates to the completion of IPT, which varies from 64.2% to 96.8% [22–28]. To stimulate TB prevention efforts, the global TB community has pledged to provide preventive treatment to 30 million people by 2022, in the first-ever United Nations High-Level Meeting (UNHLM) held in September 2018 [29].

India has been slow in adopting the policy of IPT. Even though global recommendations have been in place since 2004, India began national roll-out in 2017 only [30]. There is no published evidence from India regarding the extent of IPT implementation among PLHIV and its challenges. Generating both quantitative and qualitative evidence will be useful for national HIV and TB programs.

Hence, we aimed to assess the implementation of IPT among PLHIV, who were newly initiated on ART between January 2017 and June 2018 in two selected districts of the Karnataka State in South India. Specific objectives were to assess: (i) the proportion who were eligible for, and started on, IPT and among those initiated, the proportion who completed IPT; (ii) factors associated with initiation and non-completion of IPT, and (iii) reasons for non-initiation and non-completion from both health-care providers’ and patients’ perspectives.

## Methods

### Study design

This was an explanatory mixed-methods study with a quantitative phase (cohort study using routinely collected program data) followed by a qualitative phase (descriptive study) [31].

### Setting

#### General setting

The study was conducted in two selected districts of Karnataka State, situated in South India. Karnataka is the seventh largest Indian state by area and the eighth largest state by population (~61 million). About 60% of people live in rural areas and nearly 75% are literate [32,33]. Karnataka is one of the top five HIV burden states in India (0.47% prevalence in 15–49 years age group) with about 0.25 million PLHIV [34]. Karnataka provides HIV care through 64 ART centers, 85 Link ART Plus and 109 Link ART centers situated in 30 districts. The initial assessment and ART initiation are done at the main ART centers. Once clinically stable, PLHIV may continue ART at peripheral health facilities designated as the Link ART and Link ART Plus centers. But TB prevention services which include dispensing of isoniazid (INH) tablets are done only at the main ART centers [30,35].

#### Specific setting

We conducted this study in two selected districts – Kolar (one ART center) in the southern region and Belgaum (six ART centers) in the northern region of the State. We selected these two districts (one in each region) based on convenience and feasibility.

As per the new WHO 2016 guidelines, the National AIDS Control Organization (NACO) in India follows the ‘test and treat’ policy wherein all newly registered PLHIV are to be started on ART irrespective of CD4 count and clinical staging [4].

At ART centers, all PLHIV are screened for TB at every visit, using the 4-symptom (4S) complex which includes cough of any duration, fever, weight loss, and night sweats among adults. In children, the 4S complex includes current cough, fever, poor weight gain, and history of contact with a TB case. This screening rule has a negative predictive value of 97.7% at 5% TB prevalence in PLHIV [36]. The 4S screening is documented every month using a stamp (4S negative with Blue stamp and 4S positive with Red stamp) or by the medical officer in writing in the patient treatment record (‘White Card’) kept at the ART center and also in the patients ‘green’ book. If the patient is found to be 4S positive, then he/she is tested for TB using Xpert MTB/RIF assay and if diagnosed as TB, is started on anti-TB treatment. Patients with no TB on evaluation are treated symptomatically and a decision on IPT is made in the next visit.

According to the national guidelines, all PLHIV (new and old) must be given 6 months of IPT if 4S negative, they do not have TB and there are no contraindications (like jaundice or peripheral neuropathy) [30]. The 4S screening is usually done by the counselor or the staff nurse at the ART center, which is finally confirmed and a decision to initiate IPT is made by the medical officer of the ART center.

IPT regimen for adults and adolescents is: Isoniazid 300 mg and Pyridoxine 50 mg (Vitamin B6) per day for 6 months and for children above 12 months, it is: Isoniazid 10mg/kg and Pyridoxine 25 mg (Vitamin B6) per day for 6 months. In children <12 months of age, IPT is recommended by the WHO if the child is in contact with a case of TB [5]. IPT drugs are dispensed monthly along with the ART drugs for the patients and are documented in the opportunistic infection (OI) prophylaxis column (Section 13) of the patient white card. While receiving IPT, PLHIV are screened every month for the development of TB symptoms and evaluated. If found to have TB (termed ‘breakthrough TB’), IPT is stopped and a full course of anti-TB treatment is started. Previous history of TB treatment is not a contraindication for starting IPT. IPT is given only once during the lifetime of PLHIV [30].

The responsibility of procuring and ensuring an uninterrupted supply of the drugs required for IPT lies with the national TB program. The procurement is done centrally at the national level and supplied to states and districts. At the district level, the supply is from the District AIDS Control Society to the respective ART Centers, where the drugs are dispensed to the PLHIV. All the drugs are provided free of charge to the patients.

### Study population

#### Quantitative

All newly registered PLHIV started on ART in the two selected districts of Karnataka (Belgaum and Kolar) from 1 January 2017 to 30 June 2018 were included.

#### Qualitative

Health-care providers involved in IPT implementation and selected PLHIV in the two selected districts were interviewed. The providers included the ART staff – seven counselors, six medical officers, six staff nurses, one district tuberculosis officer (DTO), and two district TB-HIV supervisors. A purposive sampling method was used so that diverse cadres of staff were represented.

Purposive sampling was done at the patient level also so as to include patients with different outcomes (five who completed IPT and three who had stopped IPT). Data were collected during February – June 2019.

### Data variables, sources of data and data collection

#### Quantitative

Data on socio-demographic and clinical factors like age, sex, marital status, education status, occupation, initial CD4 count, type of ART regimen, WHO clinical stage, weight, height, alcohol use, tobacco use, number of monthly IPT collections was captured using a structured, pre-tested and validated, data collection proforma by the principal investigator. The source of data was the patient’s ‘white card’ maintained at the respective district ART centers.

#### Qualitative

In-depth interviews were conducted face-to-face using an open-ended interview guide. Separate interview guides were used to interview patients (n = 8) and health-care providers (n = 22). All the patient interviewees were adults and four were males. Of the eight patients interviewed, five had completed IPT and three had stopped IPT. Patients who visited the ART center on the day interviewer visited the center and belonged to the study cohort chosen for quantitative study were approached for an in-depth interview. Similarly, the health-care providers of different cadres available on the day of data collection were approached for interviews. All the participants who provided written consent were included. The number of participants interviewed was guided by the saturation of findings. All the interviews were scheduled at the ART centers in a separate cabin to maintain privacy and confidentiality.

Interviews were conducted by the principal investigator, who is a male medical doctor (MD Community Medicine), fluent in both the local language (Kannada) and English, familiar with the local context and trained in qualitative research. The interviewer is working in a medical college and is not involved in program implementation. The interview guide was prepared in English, but the interviews were conducted in the local language Kannada. Because the interviewer was bilingual in both Kannada and English, we did not translate the interview guide into Kannada. Although the interview was conducted in Kannada, the interviewer took notes (translated in English) while conducting the interview and this was transcribed immediately after the interview. We used ‘member checking’ at the end of each interview to ensure that we understood the interviewee’s responses correctly.

The proportion of IPT initiation and completion was calculated for the ART center before the interview in that center. During the interview of health-care providers, these proportions were used to highlight the gaps in initiation and completion rates in their respective centers. This enabled us to overcome the usual denial of any such deficiencies by the health-care providers. Also, insights from the quantitative analysis regarding deficiencies in eligibility evaluation, recording of test results and the timing of IPT initiation, were used to customize our interview schedule to explore reasons specific to each ART center. Similarly, in interviews with patients, we were able to customize our probes based on their demographic profiles in the patient records.

### Data entry and analysis

#### Quantitative

Data were initially entered using EpiData version 3.1 (EpiData Association, Odense, Denmark) and later exported to Stata 11.0 (StataCorp LP, College Station, TX, USA) for analysis. Demographic, clinical and treatment characteristics were summarized as percentages.

PLHIV, who had a documented 4S negative screening or those without a diagnosis of TB during the study period, were considered IPT eligible. People, with at least one documented monthly collection of IPT by the censor date (15 June 2019), were considered as ‘IPT initiated’ and those with six documented monthly collections were considered as ‘IPT completed’. The reasons for non-completion of IPT were summarized (Stopped IPT due to medical or other reasons, breakthrough TB, lost to follow-up, death, and transfer out). We used the Asian body mass index cut-offs to define overweight and obesity [37,38].

To assess the independent effect of socio-demographic and clinical characteristics of patients in IPT initiation and non-completion, a modified Poisson regression with robust variance estimates was used and adjusted risk ratios with 95% confidence intervals (CI) were calculated. We checked for variance inflation factor (VIF) to assess collinearity and those variables showing VIF more than 10 were removed from the final model. A ‘p’ value of ≤0.05 was considered to be statistically significant.

#### Qualitative

Manual descriptive thematic analysis of the transcripts was done by the principal investigator and co-investigator to identify themes under the broad topic of ‘Reasons for non-initiation and non-completion’ [39]. We used an inductive approach to coding wherein documented interview texts were read line by line thoroughly and codes were assigned to paragraphs or segments of texts relevant to the broad topic of interest. The codes conveying a particular meaning were grouped into a category. A third author reviewed the analysis and any disagreements between researchers were resolved by discussion. The findings are reported as per the ‘Consolidated Criteria for Reporting Qualitative Research’ (COREQ) [40].

## Results

### Quantitative

Of the total of 4474 PLHIV newly initiated on ART, treatment cards were not available for 454 (10%) patients and hence excluded from analysis. Patients excluded were similar to those included with respect to age and sex, but had lower CD4 counts and were more likely to be dead or lost to follow-up (LFU) (Supplementary table 1).

The mean age (SD) of study participants was 36.4 (13.3) years and 2142 (53%) were females. Among the patients, 1731 (43.1%) had low body mass index and 1278 (31.8%) had their baseline CD4 counts less than 200. The majority of the patients (85%) received a fixed-dose combination ART regimen consisting of tenofovir, lamivudine, and efavirenz (TLE) (Tables 1 and 2).

**Table 1.** Socio-demographic characteristics of newly registered PLHIV from January 2017 to June 2018 at ART centers in two selected districts of Karnataka, India

Characteristics	N	(%)
**Total**	**4020**	**(100)**
**Age in years**		
Less than 15	239	(6.0)
15–24	370	(9.2)
25–34	1075	(26.7)
35–44	1202	(29.9)
45–54	744	(18.5)
55–64	294	(7.3)
≥65	96	(2.4)
**Gender**		
Male	1863	(46.3)
Female	2141	(53.3)
Others	16	(0.4)
**Education^a^**		
Illiterate	1660	(41.3)
Primary School	1253	(31.2)
Secondary School	739	(18.4)
College and above	342	(8.5)
Not recorded	26	(0.6)
**Occupation**		
Employed	2866	(71.3)
Student	235	(5.9)
Housewife	732	(18.2)
Unemployed/retired	95	(2.3)
Not recorded	92	(2.3)
**Marital Status^a^**		
Married/live-in	2420	(60.2)
Unmarried	496	(21.8)
Widow	876	(12.3)
Divorced/separated	224	(5.6)
Not recorded	4	(0.1)
**Socio-economic class^b^**		
Class I	65	(1.6)
Class II	265	(6.6)
Class III	658	(16.4)
Class IV	1298	(32.3)
Class V	1336	(33.2)
Not recorded	398	(9.9)
**District**		
Belgaum	3319	(82.6)
Kolar	701	(17.4)

^a^As categorized and recorded in the ART register.

^b^As per BG Prasad Classification.

Abbreviations: ART, antiretroviral therapy; PLHIV, people living with HIV.

**Table 2.** Clinical characteristics of newly registered PLHIV from January 2017 to June 2018 at ART centers in two selected districts of Karnataka, India

Characteristics	N	(%)
**Total**	**4020**	**(100)**
**Body Mass Index (kg/m^2^)**		
Underweight (<18.5)	1731	(43.1)
Normal (18.5–22.99)	1530	(38.0)
Overweight (23.0–24.99)	349	(8.7)
Obese (≥25.0)	366	(9.1)
Not recorded	44	(1.1)
**CD4 cell count (cells/mm^3^)**		
Less than 200	1278	(31.8)
200–349	788	(19.6)
350–500	747	(18.6)
More than 500	1137	(28.3)
Not recorded	70	(1.7)
**WHO Clinical Staging^a^**		
Stage I	1814	(45.1)
Stage II	1773	(44.1)
Stage III	374	(9.4)
Stage IV	50	(1.2)
Not recorded	9	(0.2)
**Functional status^a^**		
Working	3600	(89.6)
Ambulatory	243	(6.0)
Bedridden	173	(4.3)
Not recorded	4	(0.1)
**ART regimen**		
TLE regimen	3401	(84.6)
Others	268	(6.7)
Not recorded	351	(8.7)
**Anemia status^b^**		
No anemia	615	(15.3)
Mild	833	(20.7)
Moderate	2050	(51.0)
Severe	264	(6.6)
Not recorded	258	(6.4)
**Alcohol use**		
Ever user	538	(13.3)
Never user	1594	(39.7)
Not recorded	1888	(47.0)
**Smoking status**		
Ever user	309	(7.7)
Never user	1703	(42.4)
Not recorded	2008	(49.9)
**Tobacco chewing status**		
Ever user	465	(11.6)
Never user	1559	(38.8)
Not recorded	1996	(49.6)
**Entry point^a^**		
VCT (Direct walk in)	1962	(48.8)
Out/in-patient	1195	(29.7)
Private practitioner	524	(13.0)
Others	225	(5.6)
Not recorded	114	(2.9)
**ART status^a^**		
Alive on ART	3018	(75.1)
Died	466	(11.6)
Lost to follow-up	186	(4.6)
Transfer out	108	(2.7)
Opted out	230	(5.7)
Stopped (on medical advice)	12	(0.3)

^a^As categorized and recorded in the ART register.

^b^WHO classification for Asian population.

Abbreviations: ART, antiretroviral therapy; CD4, cluster of differentiation 4; PLHIV, people living with HIV; VCT, voluntary counselling and testing; TLE, tenofovir lamivudine efavirenz; WHO, World Health Organization.

#### IPT eligibility, initiation, and completion

Of the 4020 patients analysed, 3780 (94.0%, 95% CI: 93.3–94.7%) were found to be eligible for IPT initiation, and of them 1496 (39.6%, 95% CI: 38.0–41.1%) were initiated on IPT. Among those initiated on IPT, 961 (64.2%) were initiated within 1 year of starting on ART.

**Figure 1.**
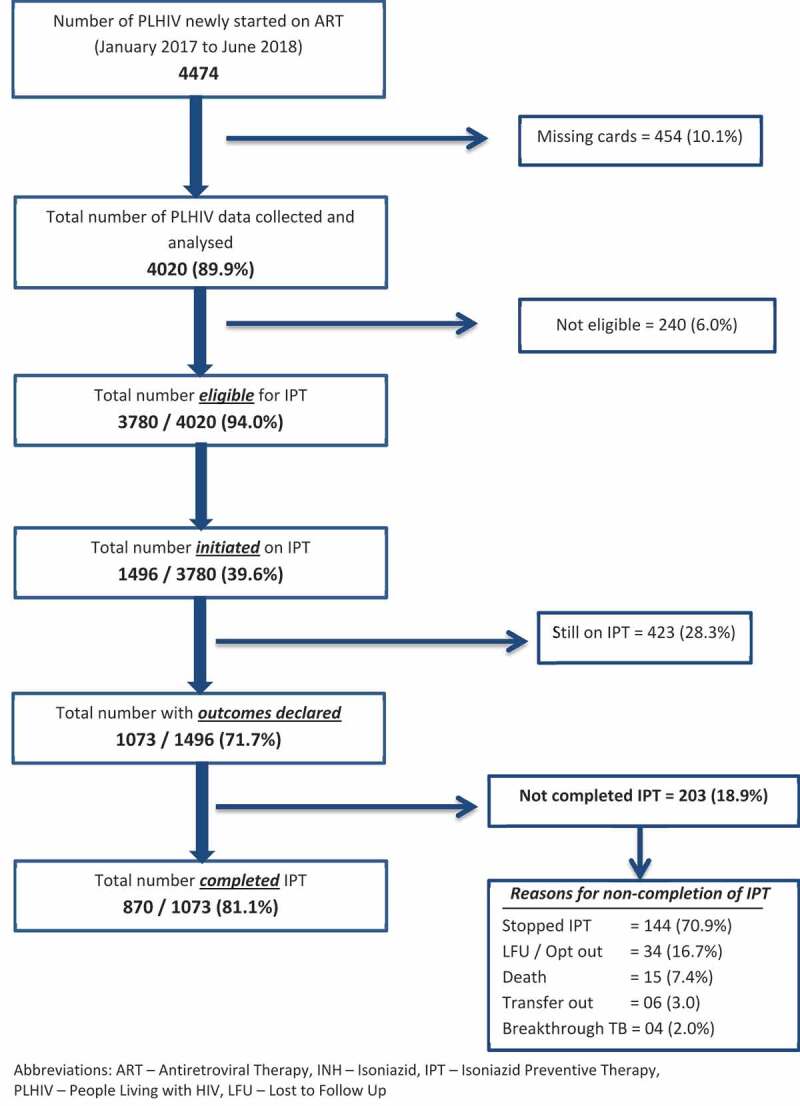
Flow diagram depicting eligibility, initiation, and completion of Isoniazid Preventive Therapy (IPT) among newly registered PLHIV from January 2017 to June 2018 at ART centers in two selected districts of Karnataka, India

Of the 1496 initiated on IPT, 423 (28.3%) were still on IPT as on censor date. Of the remaining 1073 patients in whom the outcomes for IPT were declared, 870 (81.1%, 95% CI: 78.7–83.3%) completed 6 months of IPT treatment. The key reason for non-completion was the stopping of IPT (71%) due to medical reasons such as adverse drug effects or drug stock-outs. Other reasons included LFU (17%), death (7%) and transfer out (3%). Only 4 (2%) cases developed breakthrough TB (Figure 1).

#### Factors associated with IPT initiation

Initiation of IPT was found to be significantly lower among children aged <15 years and elderly (aged ≥65 years). Initiation was also found to be higher among females, those with formal education compared to illiterate and obese individuals compared to underweight. The rates also varied significantly across the ART centers (Table 3).

**Table 3.** Socio-demographic and clinical factors associated with IPT initiation among eligible PLHIV started on ART from January 2017 to June 2018 at ART centers in two selected districts of Karnataka, India

Characteristics	Eligible for IPT, N	Initiated on IPT, n (%)^c^	Unadjusted RR (95% CI)	Adjusted RR (95% CI)^d^
**Total**	**3780**	**1496 (39.6)**		
**Age in years**				
Less than 15	229	67 (29.3)	0.7 (0.6–0.8)	0.7 (0.5–0.9)^e^
15–24	357	144 (40.3)	0.9 (0.8–1.1)	0.9 (0.8–1.0)
25–34	1019	404 (39.7)	0.9 (0.8–1.0)	0.9 (0.8–1.0)
35–44	1126	481 (42.7)	1	1
45–54	690	270 (39.1)	0.9 (0.8–1.0)	1.0 (0.9–1.1)
55–64	271	106 (39.1)	0.9 (0.8–1.1)	1.0 (0.8–1.1)
≥65	88	24 (27.3)	0.6 (0.5–0.9)	0.7 (0.5–1.0)^e^
**Gender**				
Male	1705	665 (39.0)	1	1
Female	2060	821 (39.9)	1.0 (0.9–1.1)	1.1 (1.0–1.2)^e^
Others	15	10 (66.7)	1.7 (1.2–2.5)	1.3 (1.0–1.7)
**Education^a^**				
Illiterate	1566	537 (34.3)	1	1
Primary School	1183	499 (42.2)	1.2 (1.2–1.4)	1.2 (1.1–1.3)^e^
Secondary School	688	313 (45.5)	1.3 (1.2–1.5)	1.2 (1.0–1.3)^e^
College and above	317	135 (42.6)	1.2 (1.1–1.4)	1.1 (1.0–1.3)
Not recorded	26	12 (46.2)	1.4 (0.9–2.1)	1.2 (0.8–1.9)
**Occupation**				
Employed	2666	1064 (39.9)	1	1
Student	227	83 (36.6)	0.9 (0.8–1.1)	1.1 (0.9–1.4)
Housewife	711	283 (39.8)	1.0 (0.9–1.1)	0.9 (0.8–1.0)
Unemployed/Retired	91	23 (25.3)	0.6 (0.4–0.9)	0.8 (0.5–1.1)
Not recorded	85	43 (50.6)	1.3 (1.0–1.6)	1.0 (0.8–1.2)
**Marital status^a^**				
Married/live-in	2268	931 (41.1)	1	1
Unmarried	468	170 (36.3)	1.0 (0.9–1.1)	1.0 (0.9–1.1)
Widow	833	326 (39.1)	0.9 (0.8–1.0)	1.0 (0.9–1.2)
Divorced/separated	207	68 (32.9)	0.8 (0.7–1.0)	0.9 (0.7–1.1)
Not recorded	4	1 (25.0)	0.6 (0.1–3.3)	0.5 (1.1–2.2)
**Socio-economic class^b^**				
Class I	63	33 (52.4)	1.5 (1.2–1.9)	1.0 (0.7–1.2)
Class II	250	122 (48.8)	1.4 (1.2–1.6)	1.0 (0.8–1.1)
Class III	617	274 (44.4)	1.3 (1.1–1.4)	1.0 (0.9–1.1)
Class IV	1204	479 (39.8)	1.2 (1.0–1.3)	1.0 (0.9–1.1)
Class V	1263	437 (34.6)	1	1
Not recorded	383	151 (39.4)	1.1 (1.0–1.3)	0.9 (0.8–1.1)
**ART centers**				
ART center 1	671	212 (31.6)	1	1
ART center 2	380	189 (49.7)	1.6 (1.4–1.8)	1.4 (1.2–1.7)^e^
ART center 3	337	79 (23.4)	0.74 (0.6–0.9)	0.7 (0.6–0.9)^e^
ART center 4	631	180 (28.5)	0.9 (0.8–1.1)	0.9 (0.7–1.0)
ART center 5	359	129 (35.9)	1.1 (1.0–1.4)	1.1 (0.9–1.3)
ART center 6	768	463 (60.3)	1.9 (1.7–2.2)	1.9 (1.7–2.2)^e^
ART center 7	634	244 (38.5)	1.2 (1.1–1.4)	1.1 (1.0–1.3
**Body Mass Index (kg/m^2^)**^$^****				
Underweight (<18.5)	1572	590 (37.5)	1	1
Normal (18.5–22.99)	1462	593 (40.6)	1.1 (1.0–1.2)	1.1 (1.0–1.2)
Overweight (23.0–24.99)	341	147 (43.1)	1.2 (1.0–1.3)	1.1 (1.0–1.3)
Obese (≥25.0)	364	162 (44.5)	1.2 (1.0–1.4)	1.1 (1.0–1.3)^e^
Not recorded	41	4 (9.8)	0.3 (0.1–0.7)	0.2 (0.1–0.6)^e^
**CD4 cell count (cells/mm^3^)**				
Less than 200	1142	493 (43.2)	1.1 (1.0–1.3)	1.1 (1.0–1.2)
200–349	745	294 (39.5)	1.0 (0.9–1.2)	1.0 (0.9–1.1)
350–500	719	272 (37.8)	1.0 (0.9–1.1)	0.9 (0.8–1.0)
More than 500	1114	424 (38.1)	1	1
Not recorded	60	13 (21.7)	0.6 (0.4–0.9)	0.5 (0.3–0.8)^e^
**WHO Clinical Staging^a^**				
Stages I and II	3461	1395 (40.3)	1.3 (1.1–1.5)	0.9 (0.7–1.0)
Stages III and IV	311	100 (32.2)	1	1
Not recorded	8	1 (12.5)	0.4 (0.1–2.5)	0.5 (0.1–2.4)
**Functional status^a^**				
Working	3452	1399 (40.5)	1.4 (1.1–1.8)	-
Ambulatory	187	57 (30.5)	1.0 (0.7–1.5)	-
Bedridden	137	40 (29.2)	1	-
Not recorded	4	0 (0.0)	-	-

^a^As categorized and recorded in the ART register.

^b^As per BG Prasad Classification.

^c^Row Percentage.

^d^The ‘Functional status’ was taken out from the final model as the variance inflation factor was greater than 10.

^e^p value <0.05.

^$^WHO Classification for Asian population.

Abbreviations: ART, antiretroviral therapy; CD4, cluster of differentiation 4; CI, confidence interval; IPT, isoniazid preventive therapy; PLHIV, people living with HIV; RR, relative risk; TLE, tenofovir lamivudine efavirenz; WHO, World Health Organization.

#### Factors associated with IPT non-completion

In the adjusted analysis, the completion rates varied significantly across the ART centers (Table 4).

**Table 4.** Socio-demographic and clinical factors associated with IPT non-completion among PLHIV started on IPT from January 2017 to June 2018 at ART centers in two selected districts of Karnataka, India

Characteristics	Initiated on IPT, N	Not Completing IPT, n (%)^c^	Unadjusted RR (95% CI)	Adjusted RR (95% CI)
**Total**	**1073**	**203 (18.9)**		
**Age in years**				
Less than 15	40	6 (15.0)	0.9 (0.4–1.9)	0.5 (0.2–1.4)
15–24	103	22 (21.4)	1.2 (0.8–1.9)	0.8 (0.5–1.4)
25–34	289	66 (22.9)	1.3 (1.0–1.8)	1.0 (0.8–1.4)
35–44	356	61 (17.1)	1	1
45–54	190	33 (17.4)	1.0 (0.7–1.5)	1.1 (0.8–1.7)
55–64	77	11 (14.3)	0.8 (0.5–1.5)	0.9 (0.5–1.5)
≥65	18	4 (22.2)	1.3 (0.5–3.1)	1.1 (0.5–2.3)
**Gender**				
Male	479	85 (17.8)	1	1
Female	586	117 (20.0)	1.1 (0.9–1.5)	1.2 (0.9–1.6)
Others	8	1 (12.5)	0.7 (0.1–4.5)	0.3 (0.1–1.6)
**Education^a^**				
Illiterate	393	64 (16.3)	1	1
Primary school	354	78 (22.0)	1.4 (1.0–1.8)	1.1 (0.8–1.5)
Secondary school	225	42 (18.7)	1.1 (0.8–1.6)	0.8 (0.6–1.2)
College and above	95	19 (20.0)	1.2 (0.8–1.9)	0.8 (0.5–1.3)
Not recorded	6	0 (0.0)	-	-
**Occupation**				
Employed	779	133 (17.1)	1	1
Student	52	10 (19.2)	1.1 (0.6–2.0)	0.9 (0.4–1.9)
Housewife	196	42 (21.4)	1.3 (0.9–1.7)	1.0 (0.7–1.4)
Unemployed/retired	15	5 (33.3)	2.0 (0.9–4.1)	1.7 (0.8–3.6)
Not recorded	31	13 (41.9)	2.5 (1.6–3.8)	1.1 (0.7–1.8)
**Marital status^a^**				
Married/live-in	660	122 (18.5)	1	1
Unmarried	236	49 (20.8)	1.1 (0.8–1.5)	1.1 (0.8–1.5)
Widow	120	30 (25.0)	1.4 (1.0–1.9)	1.5 (1.0–2.3)
Divorced/separated	56	2 (3.4)	0.2 (0.1–0.8)	0.3 (0.1–1.2)
Not recorded	1	0 (0.0)	-	-
**Socio-economic class^b^**				
Class I	22	8 (36.4)	3.0 (1.6–5.5)	1.2 (0.6–2.4)
Class II	87	24 (27.6)	2.2 (1.4–3.5)	1.1 (0.7–1.7)
Class III	188	43 (22.9)	1.9 (1.3–2.7)	1.0 (0.7–1.6)
Class IV	345	52 (15.1)	1.2 (0.8–1.8)	0.8 (0.6–1.3)
Class V	324	40 (12.4)	1	1
Not recorded	107	36 (33.6)	2.7 (1.8–4.0)	1.1 (0.7–1.7)
**ART centers**				
ART center 1	124	6 (4.8)	1	1
ART center 2	141	14 (9.9)	2.1 (0.8–5.2)	2.6 (1.0–6.8)
ART center 3	44	2 (4.6)	0.9 (0.2–4.5)	0.6 (0.1–4.5)
ART center 4	161	14 (8.7)	1.8 (0.7–4.5	1.8 (0.7–4.8)
ART center 5	113	8 (7.1)	1.5 (0.5–4.1)	1.6 (0.6–4.6)
ART center 6	328	129 (39.3)	8.1 (3.7–18.0)	8.2 (3.6–18.9)^d^
ART center 7	162	30 (18.5)	3.8 (1.7–8.9)	4.4 (1.8–10.7)^d^
**Body mass index (kg/m^2^)**^$^****				
Underweight (<18.5)	435	88 (20.3)	1	1
Normal (18.5–22.99)	421	79 (18.8)	0.9 (0.7–1.2)	0.9 (0.7–1.2)
Overweight (23.0–24.99)	100	16 (16.0)	0.8 (0.5–1.3)	0.7 (0.5–1.2)
Obese (≥25.0)	116	19 (16.4)	0.8 (0.5–1.3)	0.7 (0.5–1.2)
Not recorded	1	1 (100.0)	-	-
**CD4 cell count (cells/mm^3^)**				
Less than 200	390	59 (15.1)	1	1
200–349	215	43 (20.0)	1.3 (0.9–1.9)	1.3 (0.9–1.8)
350–500	185	44 (23.8)	1.6 (1.1–2.3)	1.4 (1.0–2.0)
More than 500	276	54 (19.6)	1.3 (0.9–1.8)	1.3 (0.9–1.8)
Not recorded	7	3 (42.9)	2.8 (1.2–6.9)	2.8 (1.0–7.5)^d^
**WHO Clinical Staging^a^**				
Stages I and II	999	190 (19.0)	1	1
Stages III and IV	73	13 (17.8)	1.0 (0.6–1.6)	1.3 (0.8–2.0)
Not recorded	1	0 (0.0)	-	-
**Functional status^a^**				
Working	1012	188 (18.6)	1	1
Ambulatory	38	7 (18.4)	1.0 (0.5–2.0)	1.0 (0.6–2.0)
Bedridden	23	8 (34.8)	1.9 (1.1–3.3)	1.2 (0.6–1.8)

^a^As categorized and recorded in the ART register.

^b^As per BG Prasad Classification.

^$^WHO Classification for Asian population.

^c^Row Percentage.

^d^p value <0.05.

Abbreviations: ART, antiretroviral therapy; CD4, cluster of differentiation 4; CI, confidence interval; IPT, isoniazid preventive therapy; PLHIV, people living with HIV; RR, relative risk; TLE, tenofovir lamivudine efavirenz; WHO, World Health Organization.

### Qualitative: reasons for IPT non-initiation and non-completion

**Figure 2.**
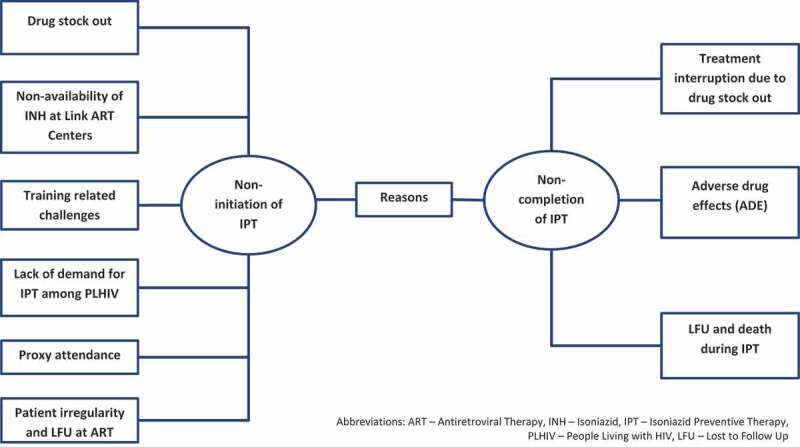
Reasons for non-initiation and non-completion of IPT among newly registered PLHIV during January 2017 and June 2018 at ART centers in two selected districts of Karnataka, India as perceived by patients (n = 8) and health-care providers (n = 22)

A total of 31 codes were deduced from the transcripts and categorized into two broad themes as summarized in Figure 2.

#### Reasons for non-initiation of IPT

The codes related to this broad theme were grouped into six categories as described below.

##### Drug stock out

The health-care providers at the ART center reported that frequent drug stock-out was the major reason for not initiating IPT.

It was always drug shortage … as we give ART tablets to the patients regularly, if these drugs [IPT] are also supplied sufficiently then we will give IPT tablets to the patients along with ART! (30 year old male ART counsellor)

Adapting to this situation, the providers reported that they started prioritizing patient subgroups for IPT initiation based on arbitrary and varied criteria including CD4 count, regularity of visits, and adherence to ART. Providers also mentioned about ear-marking drugs for the entire 6-month course of IPT for each patient, in order to avoid interruption of IPT once started, and prevent the development of isoniazid resistance even if this meant not initiating IPT in several patients.

There were repeated stock outs! … Then we set some criteria … .like … patients having less than 250–300 cell count, at least for them we thought of giving for complete six months. So we continued with this criteria sir! (35 year old male TB-HIV supervisor)

##### Non-availability of isoniazid at link ART centers

The health-care providers noted that there were many patients receiving care at Link ART and Link ART plus centers who visit the parent ART center only once every 6 months. Initiating such patients on IPT was a challenge as isoniazid was not available at link centers.

At Link ART side they will collect ART tablets. Isoniazid also they can collect there … But, that is not allowed now in program its only from the ART center … (District Tuberculosis Officer)

##### Training-related challenges

Most of the health-care providers felt that training was not given to all cadres of staff. Also, those who had received training were either not working currently in the ART center or were not involved in the delivery of IPT. Some providers were not sure if IPT should be given to children and pregnant women.

Training was given to Staff Nurse and Pharmacist … . but both did not initiate IPT … so I think training should be given to counsellor … There is need sir … They didn’t give … (35 year old male ART counsellor)

##### Lack of demand for IPT among PLHIV

The health-care providers perceived that patients do not value the preventive effect of IPT because of low perceived risk, lack of symptoms and fear of adding to the pill burden.

Because people will not know that what is this prophylaxis. all will feel healthy and if we tell about TB then they think I am good why to take these tablets? (33 year old male medical officer)

They also expressed that there was no information, education and communication materials to educate PLHIV and social campaigns to generate demand for IPT among patient groups.

Moreover there are no IEC (Information Education Counselling) materials regarding IPT! We have for others like TB, PPTCT (Prevention of Parent To Child Transmission), for condom and all it is there! At least by seeing the IEC material … educated people will read and will know for what IPT strips are given for! (35 year old male ART staff nurse)

##### Proxy attendance

The health-care providers reported that in many instances, patients send their caregivers or attenders on their behalf for ART refill. Thus, patients were not available to screen for 4S and assess eligibility for IPT initiation.

Instead of patient the caretaker will come … but we can’t send tablets with them … … so we start IPT only when patient comes to the center for screening of 4S. (28 year old male ART counsellor)

##### Patient irregularity and LFU at ART center

The health-care providers felt that patient irregularity and also high LFU rate played a major role in the non-initiation of IPT at their respective ART centers.

IPT initiation is only 50% … another 30% to 40% people who are eligible are not coming to ART center and they are not taking ART also and they are not taking IPT also … that’s the matter (District Tuberculosis Officer)

The health-care providers reported alcoholism in patients, poor literacy, shortage of staff, and high patient load as some of the other reasons that caused a delay in the initiation of IPT.

#### Reasons for non-completion of IPT

The codes related to this broad theme were grouped into three categories as described below.

##### Treatment interruption due to drug stock out

The health-care providers felt the major reason for non-completion was a frequent interruption of drug supplies.

In the beginning, as we received the drugs we started treatment for everyone, A to Z to all the patients … But after two three months there was drug shortage and we weren’t issued sufficient stock, so again discontinuation occurred and treatment was stopped (36 year old male staff nurse)

##### Adverse drug effects (ADE)

Both providers and patients (who discontinued the treatment) opined that ADE was an important reason for discontinuation of IPT.

Actually had giddiness, vomiting, nausea, my appetite reduced, indigestion … sleep was also more … . Later when I came for the next visit I told them that I had vomiting whenever I took these tablets and then the doctor said “it’s ok you need not take” … (25 year old female patient who discontinued IPT)

There was a shortage of pyridoxine too, a drug prescribed to prevent peripheral neuropathy caused by Isoniazid.

Mostly because of side effects … neuropathies … and we dont have pyridoxine. we are not giving that to them … (55 year old male medical officer)

##### LFU and death during IPT

Another key reason for non-completion in the view of health-care providers was LFU and death.

Missed cases … LFU is more … . Like one month they come and then they won’t come … (38 year old female staff nurse)

Other reasons listed for non-completion included pill burden, lack of family or social support and also access to social protection schemes.

## Discussion

As one of the first studies from India evaluating the IPT uptake, completion and implementation challenges among PLHIV in routine program settings, the work adds to the global evidence on this topic. The findings are important. First, the IPT initiation was sub-optimal in only two out of five PLHIV initiated on IPT. Second, the completion rate among those initiated was promising with about four in five completing the 6-month course. Third, the reasons for deficiencies in initiation and completion were mostly attributed to frequent drug stock-outs, proxy attendance of patients at ART centers, lack of training and ADEs associated with IPT.

The combined picture of ‘low IPT initiation and high completion rates’ seen in our study mirrors the findings of studies conducted in other countries [22,24,26,28]. The IPT initiation rates reported here are on par with the global estimate (36%) and much higher than the Indian national estimate (10%) [1]. Potential reasons for this difference include: (i) systematic and rigorous assessment of the patient records; (ii) relatively long follow-up periods (ranging from 12 to 30 months), and (iii) the study being conducted in Karnataka State, which is one of the relatively well-performing states in TB-HIV co-management.

We believe there were some factors that might have overestimated or underestimated IPT initiation and completion rates. First, we failed to trace the treatment cards of about 10% of the patients who had relatively higher rates of LFU and death and thus might have had lower IPT initiation and completion rates. Excluding them might have marginally overestimated our IPT initiation and completion rates. Second, due to deficiencies in the documentation of 4S screening results and other potential contra-indications (jaundice, elevated liver enzymes, peripheral neuropathy, etc.) for starting IPT, we defined those without TB as eligible. Hence, we might have overestimated the proportion eligible for IPT and thus underestimated the IPT initiation rates. Third, we operationally defined IPT initiation and completion based on documented monthly drug collections. However, ‘drug collection’ is not equivalent to ‘drug consumption’ and there was no assessment of IPT adherence based on pill count or refill as being done for ART. This might have overestimated the IPT completion rates. The net effect of these different factors on our estimates is unclear.

There were some other limitations. The study was conducted only in two districts and hence the findings may not be generalizable to other districts and states within India. Since we had no information on the date of eligibility, we could not assess the time to IPT initiation from eligibility and conduct a survival analysis. Similarly, information on the regularity of IPT collections and the duration it took from initiation to completion could not be assessed. Also, as the reasons for stopping IPT were captured as a single option in the ‘white cards’, the disaggregation with respect to stopping due to stock-outs and due to adverse drug effects was not possible. In spite of all the efforts in conducting qualitative interviews in local language and transcribing in English and finally confirming the content through participant validation, we might have lost some information due to translation. Finally, we failed to explore the reasons for IPT non-initiation from patients’ perspectives. While we achieved saturation in capturing providers’ views, we may not have achieved the same in capturing patients’ voices.

The study had several strengths. First, we used a mixed-methods design which enabled us to not only assess the extent of implementation of IPT but also assess the reasons for gaps in implementation. Since the qualitative assessment was done by a medical college faculty who is not involved in program implementation, this might have helped in reducing the bias and subjectivity in the analysis. Second, we conducted a cohort-wise analysis using data collected in routine program settings and thus the findings reflect the on-the-ground realities. Third, the large sample size enabled us to precisely estimate initiation and completion rates and associated factors. Fourth, we adhered to STrengthening the Reporting of OBservational studies in Epidemiology (STROBE) and COREQ guidelines for reporting the study findings [40,41].

There are some important programmatic implications arising out of our study.

First, the key reason identified for both non-initiation and non-completion of IPT was drug stock-outs. This seems to be the major barrier for IPT implementation globally and has been reported elsewhere too [23,42]. These drug stock-outs are not unique to IPT but also a feature for ART and other drugs and thus forms a part of health system challenges faced in all low- and middle-income countries [43–46]. In our study, the health-care providers adapted to the situation by prioritizing some patient groups and earmarking six-monthly courses for each patient started on IPT. The practice ensured that there was no interruption in IPT once started, the providers believed that this would reduce the emergence of isoniazid resistance. But, this came with a cost of lower rates of IPT initiation. This might explain the overall picture of ‘lower IPT initiation and high completion’. These findings call for strengthening the central procurement and supply chain management to ensure an uninterrupted supply of IPT drugs and strengthening the decentralized delivery of IPT. We also recommend that the district TB office must be empowered to procure isoniazid locally in order to meet the challenge of drug stock-outs.

Second, IPT is currently dispensed only at the ART centers, whereas most of the clinically stable patients collect their ART drugs from ‘Link ART’ centers. Patients managed at ‘Link ART’ centers visit the parent ART centers only once in 6 months and are rarely initiated on IPT. Even if initiated, the patients are required to visit ART centers to collect IPT drugs. The program needs to revise the strategy and make IPT available at ‘Link ART’ centers too, to increase the coverage and completion of IPT.

Third, proxy attendance for patients to collect ART drugs had led to poor initiation of IPT. This reason was not identified in other studies and could be a unique feature of our settings. It is an important issue in relation to the initiation of IPT as the presence of patients is essential to screen for IPT eligibility. Also, practical programmatic and patient-related considerations mean that it is not always possible to start IPT along with ART at the first visit and thus may miss out on a large number of patients who use their proxies to collect ART refills and visit ART centers only once in 6 months. Another consideration is that the patients’ educational levels may influence their attendance for drug collection. For example, we found that, compared to those who were illiterate, patients who had formal schooling had a higher chance of IPT initiation.

Fourth, we found in quantitative analysis that the children and elderly were less likely to receive IPT. This might be related to a lack of clarity among providers on the initiation of IPT for children and some other subgroups such as pregnant women. There are separate tablets with pediatric dosage available for children in the program but not many health-care providers were aware of this. This highlights the deficiencies in the training process. As emphasized by the providers, all ART staff who are involved in IPT implementation need to be trained and there must be periodic refresher training on IPT.

Fifth, the IPT initiation and completion rates varied across ART centers. Thus, programmatic issues like deficiency of adequate human resource and drug supply at each center determine the extent of IPT implementation in each center. This could also be related to financial barriers in accessing ART centers. As stated by the providers, the policy of providing travel support has now been made conditional and is only available for selected patient subgroups. This needs to be reinstituted for all the patients as it impacts adherence to HIV care in general.

Sixth, adverse drug effects were a major reason for non-completion. This was compounded by the poor nutritional status of the patients and the shortage of essential drugs such as pyridoxine required for preventing peripheral neuropathy. The eligibility assessment needs to be strengthened to rule out pre-existing morbidities like hepatitis and alcohol use which may contribute to the occurrence of ADEs. While it is advisable to take isoniazid on an empty stomach, patients with gastrointestinal ADEs may be counseled to consume drugs along with food for some days until one gets used to the drug. There has been great progress in reducing the duration of the TB preventive therapy using newer regimens such as isoniazid and rifapentine, either once a week for 12 weeks or once daily for 1 month [47,48]. This is likely to reduce ADEs and increase completion rates. But, this regimen is currently expensive and not easily available as rifapentine is not yet approved for use by the drug controller general of India. These issues need to be considered by the national TB and HIV programs in India before adoption.

Finally, there were challenges in collecting data for evaluating routine TB preventive services and this could be addressed partially by using mobile applications developed by WHO which can be adapted to local settings [49].

## Conclusion

IPT initiation was suboptimal but IPT completion was more encouraging in two selected districts of Karnataka, South India. The reasons for non-initiation and non-completion were identified and there is an urgent need to improve the procurement and supply chain management to prevent frequent drug stock-outs. Unless we address these, we are unlikely to meet the global goal of providing 30 million courses of preventive treatment by 2022 [29].

## Supplementary Material

Supplemental Material

## Paper context

This is the first study from India evaluating the IPT uptake, completion and implementation challenges among PLHIV in routine program settings. We found that the IPT initiation was suboptimal (~40%) but IPT completion was more encouraging (~80%) which is similar to other settings. The main reason for non-initiation and non-completion was frequent drug stock-outs. There is an urgent need to improve the procurement and supply chain management of isoniazid.
