# Supplementary material for: To start or to complete? – Challenges in implementing tuberculosis preventive therapy among people living with HIV: a mixed-methods study from Karnataka, India
Source: Glob Health Action. 2020 Jan 15;13(1):1704540. doi: 10.1080/16549716.2019.1704540 (PMC7006687; doi:10.1080/16549716.2019.1704540)
Supplement: Supplemental Material [file ZGHA_A_1704540_SM2608.docx]

***Supplementary table 1: Comparison of study characteristics between data analysed (collected) and missing cards, N=4474***

| **Characteristics** | **Total,**  **N** | **Data analysed,**  **n (%)** | **Missing cards,**  **n (%)** | **Chi-square value, df** | **p value** |
| --- | --- | --- | --- | --- | --- |
| **Total** | **4474** | **4020 (89.9)** | **454 (10.1)** |  |  |
| **Age in years** |  |  |  |  |  |
| Less than 15 | 270 | 239 (6.0) | 31 (6.8) | 4.903, df=6 | 0.557 |
| 15 – 24 | 416 | 370 (9.2) | 46 (10.1) |  |  |
| 25 – 34 | 1177 | 1075 (26.7) | 102 (22.5) |  |  |
| 35 – 44 | 1339 | 1202 (29.9) | 137 (30.2) |  |  |
| 45 – 54 | 833 | 744 (18.5) | 89 (19.6) |  |  |
| 55 – 64 | 329 | 294 (7.3) | 35 (7.7) |  |  |
| ≥65 | 110 | 96 (2.4) | 14 (3.1) |  |  |
| **Gender** |  |  |  |  |  |
| Male | 2073 | 1863 (46.3) | 210 (46.3) | 0.667, df=2 | 0.717 |
| Female | 2382 | 2141 (53.3) | 241 (53.1) |  |  |
| Others | 19 | 16 (0.4) | 3 (0.6) |  |  |
| **CD4 cell count (cells/mm^3^)** |  |  |  |  |  |
| Less than 200 | 1451 | 1278 (31.8) | 173 (38.1) | 29.294, df=4 | <0.001 |
| 200 – 349 | 863 | 788 (19.6) | 75 (16.5) |  |  |
| 350 – 500 | 837 | 747 (18.6) | 90 (19.8) |  |  |
| More than 500 | 1233 | 1137 (28.3) | 96 (21.2) |  |  |
| Not recorded | 90 | 70 (1.7) | 20 (4.4) |  |  |
| **ART outcome status** |  |  |  |  |  |
| Alive on ART | 3249 | 3059 (76.1) | 190 (41.9) | 502.387, df=5 | <0.001 |
| Died | 523 | 440 (11.0) | 83 (18.3) |  |  |
| Lost to follow-up | 260 | 191 (4.7) | 69 (15.2) |  |  |
| Transfer Out | 199 | 105 (2.6) | 94 (20.7) |  |  |
| Opted out | 217 | 210 (5.2) | 7 (1.5) |  |  |
| Stopped (On medical advice) | 26 | 15 (0.4) | 11 (2.4) |  |  |

*Abbreviations:* ART – Antiretroviral therapy, CD4 – Cluster of Differentiation 4, df – degrees of freedom
